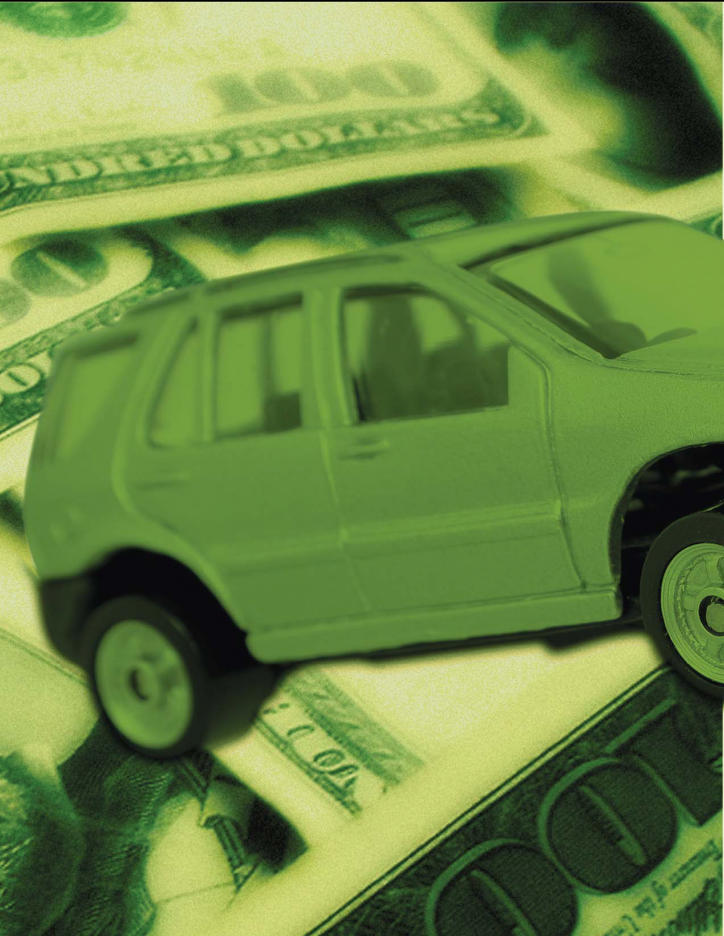# Driving Up the Cost of Clean Air

**DOI:** 10.1289/ehp.113-a246

**Published:** 2005-04

**Authors:** David C. Holzman

The internal combustion engine is a mixed blessing. The freedom of movement provided by cars and light trucks—minivans, SUVs, and pickups—is unprecedented. But these same vehicles also pose a major public health problem. It is not surprising, then, that strategies to mitigate the harmful side effects of personal transportation are a major topic of study.

In 2002, car accidents killed nearly 43,000 and incapacitated another 356,000, according to the National Highway Traffic Safety Administration. Vehicle exhaust contributes to untold numbers of hospitalizations for asthma attacks and other health problems, while car-related pollution contributes to lung diseases and heart attacks. Cars and light trucks on American roads contribute slightly less than 4% of the world’s annual burden of greenhouse gases and about 20% of the U.S. burden. Each year, car- and truck-related congestion costs the United States $63 billion in lost time and wasted fuel, according to the Texas Transportation Institute’s *2004 Urban Mobility Report*, and 5.6 billion gallons of gasoline are wasted by idling in traffic.

Much controversy swirls in the debate over how best to mitigate the harmful side effects of automotive transportation. It is somewhat helpful to sharpen the debate by appealing to economic theory, although ideals usually become badly dented when they collide with politics.

Some of these harmful side effects are what economists call “externalities,” or costs that are not incorporated into the market price. A motorist buying gasoline, for example, does not pay for the costs of the climate change or the air pollution that will result from burning that fuel, or the military cost of maintaining access to Persian Gulf oil.

Externalities have obvious solutions: figure out their costs, and incorporate them into the price. The theoretically superior strategy would be to tax individual greenhouse gas emissions and to incorporate defense costs into the price of oil specifically from the Persian Gulf (although military spending, generally viewed as a fixed rather than variable cost, is usually excluded from computations of the optimal oil or gasoline tax).

Accidents are arguably not externalities, because they are largely paid for through insurance. However, car insurance does not cover expenses such as fire and police department costs or costs related to death and pain/suffering. Furthermore, insurance is a lump-sum payment rather than a per-mile charge, so motorists likely do not consider accident costs when deciding how much to drive a given vehicle. Traffic congestion, a well-established result of motorization, is less ambiguous as an externality, although people endure part of the delays they help to create. But to the extent that accidents and congestion are externalities, they are externalities of driving, not of gasoline use, because cars can run on other fuels.

As yet, determining the cost of fuel-based externalities is a primitive art, says Mark Delucchi, a research scientist at the Institute for Transportation Studies of the University of California, Davis. “The best estimates of virtually all important external costs—air pollution, noise, accidents, congestion, and oil importing—vary by about an order of magnitude,” he wrote in the spring 2000 issue of *Access* magazine. Delucchi advocates using these estimates to inform but not determine policy.

A number of strategies have been suggested in the United States to reduce the problems created by automotive transportation. One strategy in particular—the corporate average fuel economy (CAFE) standards—has been around for several decades. The pros and cons of personal transportation and the controversy surrounding CAFE and other strategies demonstrate the need for careful thought—as well as the hazards that arise—when policy meets politics.

## High-Test CAFE

The legislation authorizing CAFE was enacted in 1975, following the 1973–1974 Arab oil embargo. CAFE standards set an average gas mileage requirement for a manufacturer’s fleet. The fuel economy of all the cars sold by a manufacturer in any given year must average 27.5 miles per gallon, and the fuel economy of all light trucks sold must average 20.7 miles per gallon. So Ford Motor Company, for example, must sell a lot of its diminutive Focuses to make up for the portly Lincolns, and the relatively small Ford Escapes must make up for the Lincoln Navigators, Ford Excursions, and other SUVs.

The goal of CAFE was to double new car fuel economy with no loss in performance, a goal that was largely achieved by 1985, according to the 2002 National Academy of Sciences (NAS) report *Effectiveness and Impact of Corporate Average Fuel Economy (CAFE) Standards*. The same might have been accomplished with a tax, but a cartel—or monopoly—is not an externality, and applying a tax on top of monopoly pricing could damage the economy. Arguably, there was no ideal solution.

CAFE is also credited with helping in 1986 to collapse prices set by the Organization of the Petroleum Exporting Countries, bringing gasoline prices down from an inflation-adjusted peak of $2.94/gallon in 1981. According to the NAS report, without CAFE, the United States would be burning an additional 2.8 million barrels per day—equivalent to about 14% of current U.S. consumption and 3% of world consumption—at a cost of $120 billion dollars annually (about 19% of the U.S. goods and services deficit for 2004).

But not everyone agrees that CAFE has been that effective at curbing fuel consumption. In the May 1997 issue of the *Journal of Regulatory Economics*, materials scientist Steven Thorpe wrote that CAFE standards may actually have contributed to a decline in the average fuel economy of the new fleet by shifting sales to less fuel-efficient vans, trucks, and SUVs.

CAFE opponents—such as Tom Walton, director of economic policy at General Motors—argue more generally that the cost of CAFE is greater than the benefits. A 9 March 2004 issue brief by the Congressional Budget Office (CBO) found that “[u]nless current estimates of the benefits of reducing gasoline consumption are significantly understated, increasing CAFE standards would not pass a benefit–cost test.”

Many opponents say that in the absence of higher gasoline prices, improved fuel economy encourages people to drive an extra 10–20% (however, according to David Greene, an environmental engineer at Oak Ridge National Laboratory, a 10% increase in fuel economy will result in only about a 1% increase in driving). Opponents further argue that the additional accidents and congestion caused by this surplus driving cost roughly $1.00 and $1.40 per gallon, respectively, at current average U.S. fleet fuel economy. These costs subsume the benefits of greater fuel economy, a combined savings on greenhouse gas effects and oil dependency of 30¢ per gallon, and on pollution of an additional 40¢ per gallon at current fleet average fuel economy.

However, Roland Hwang, vehicles policy director for the Natural Resources Defense Council, says that these arguments fail to acknowledge that fuel economy can be raised while saving consumers money. “There are private benefits to raising fuel economy—benefits which the market has not organized to capture,” he says. He points to the Energy Star labels and efficiency standards created for the appliance market by the government as an example of how to attach a social responsibility value to a product—a value that many consumers prize and thus will pay more for.

Furthermore, says Hwang, the benefits of CAFE *are* understated. Standards send a strong signal to manufacturers to organize research, which frequently pushes down the cost of incorporating advanced technologies into new models, he explains. For example, California’s stringent emissions standards led to car companies’ discovery that instead of using an expensive electrically heated exhaust-cleaning catalyst, they could move the catalyst close enough to the engine for the latter to heat it, without damaging the catalyst’s materials. The result was a substantial economic savings.

Althought the 2002 NAS report failed to endorse CAFE, it did describe a potential modification that might be more attractive—manufacturers would receive fuel economy credits for exceeding the target fleet average, and instead of meeting the target, they could buy credits from other manufacturers or the government. The prospect of selling extra credits might motivate manufacturers to boost fuel economy beyond the target—although Ian Parry, a senior fellow at Resources for the Future, notes this would be offset by other manufacturers buying credits so their fuel economy could be lower than the standard.

A carbon cap-and-trade system, also favorably described by the NAS, could extend this type of system to other fossil fuel uses as a means of reducing greenhouse gas emissions. In this scenario, the government would set a mandatory cap on total emissions, and distribute emissions rights to sellers and users, which they could trade among themselves.

## Other Measures

While CAFE has been a continuing source of controversy, the Environmental Protection Agency (EPA) Tier I regulations for tailpipe emissions have reduced emissions by about 90% since the 1970s, according to the Union of Concerned Scientists. However, automotive pollution remains a local problem in some regions, says John Millett, a spokesman for the EPA. Diesel pollution also is still a problem and will remain so for many years, says Delucchi.

The regulation strategy incorporates a political decision to protect the most vulnerable, rather than just the average person, says Hwang. The new Tier 2 regulations finalized in 1999 and the retirement of most Tier 1 automobiles by around 2030 will be the equivalent, in terms of air quality improvement, of removing 164 million cars from the road. These standards will add about $100 and $200 to the purchase price of cars and light trucks, respectively. Additionally, the removal of sulfur from gasoline adds about 2¢ per gallon at the pump.

The EPA estimates, however, that the health and environmental benefits will ultimately be worth $25.2 billion annually, at a cost to industry of $5.3 billion. The Tier 2 standards will prevent as many as 4,300 deaths, more than 10,000 cases of chronic and acute bronchitis, and tens of thousands of respiratory problems per year, the EPA estimated in its 1999 announcement of the standards.

A gasoline tax represents another strategy. In theory, a gas tax can function as a carbon tax and therefore may be appropriate for addressing the fuel-related externality of carbon dioxide emissions and the resulting climate change. But carbon dioxide is not the only vehicle emission to affect climate, and using a gasoline tax to reduce personal vehicles’ contribution to greenhouse gases could be considered wasteful, because coal-fired utilities’ contributions dwarf those of cars and trucks. In addition, a gas tax is of little use against congestion, because it doesn’t differentiate between rush hour city miles and non–rush hour or rural driving. A better method would be to tax the carbon content of all fossil fuels.

So-called feebates, endorsed by the NAS report, would provide a rebate to buyers of cars and trucks that exceed a fuel economy benchmark, and charge a fee to buyers of cars that miss the benchmark. (The federal “gas guzzler” tax represents such a fee, but lacks the complementary carrot.) Researchers at Rocky Mountain Institute (RMI) in Snowmass, Colorado, have proposed a program in which the benchmark would be reset every year to keep it revenue-neutral. The program would be size-neutral and would make money for automakers as well as consumers. “Unlike standards, feebates reward and propel continuous improvement,” according to the RMI report *Winning the Oil Endgame*.

RMI proposes complementing fee-bates with a scrap-and-replace program for low-income households, which would get what are traditionally the least efficient cars off the road, and provide poorer families with reliable, affordable personal transportation. “There is a growing consensus that limited mobility is an important missing link in a comprehensive strategy for reducing poverty,” according to *Winning the Oil Endgame*. One of two proposed mechanisms would finance highly efficient new cars through high-volume procurement and lease them to qualified low-income citizens. The corresponding scrappage of clunkers could start with the dirtiest cars.

Two other novel proposals still in the experimental stages—pay-as-you-drive (PAYD) and pay-at-the-pump (PATP)—tackle the issue from the insurance point of view. These strategies lack the poisonous aura of a tax because they would merely shift the way people make their car insurance payments without adding to the cost of driving.

By making insurance payments directly proportional to vehicle miles traveled, PAYD would act like a gas tax in boosting the motivation to reduce driving, especially for high-risk drivers. “[PAYD] insurance conveys to drivers the true costs they impose on others, and allows motorists . . . to save money by reducing these costs,” states the *Online TDM* [transportation demand management] *Encyclopedia* published by Canada’s Victoria Transport Policy Institute. “Vehicle crashes should decline even more than mileage because higher-risk motorists (who currently pay high premiums per vehicle-year) would pay higher per-mile fees, and would therefore have the greatest incentive to reduce their driving.”

The result could be to save an estimated 5,000 lives that would have been lost to crashes involving reckless drivers, according to the encyclopedia. Yet, the cost of driving would fall, on average, by $50–100 per vehicle.

Parry cautions that it would take time for insurance companies to adopt PAYD, and he advocates a tax credit for insurance companies to kick-start the market. But PAYD is no panacea. Parry, a proponent, foresees a need for separate congestion charges, which he thinks might completely replace gasoline taxes several decades hence.

PATP, on the other hand, trades PAYD’s incentive to drive less for an incentive to drive less, buy a more fuel-efficient car, or both. A basic insurance fee would be charged at the gas pump, and motorists could buy more comprehensive coverage or pay for higher-risk coverage directly from the insurance companies.

## The Best Deal

Ideally, policies should “deal directly with a problem, not indirectly,” says David O. Dapice, an economics professor from Tufts University. He suggests that a gasoline tax is no better than a Swiss army knife for dealing with safety, congestion, and air pollution. “If you are worried about congestion, tax congestion,” he says. “If you are worried about safety, set standards, or require technologies that promote safety, such as rollover standards for SUVs. If you want to deal with carbon dioxide, tax carbon.” But, he adds, “If you want to save fuel, tax fuel.”

As the controversy continues over how best to mitigate the harmful side effects of automobiles, one thing becomes abundantly clear: cars aren’t going away anytime soon, and the more we can reduce their impact on our environment and our health, the better it will be for all humankind.

## Figures and Tables

**Figure:**